# Neurotrophic effects of Botulinum neurotoxin type A in hippocampal neurons involve activation of Rac1 by the non-catalytic heavy chain (HC_C_/A)

**DOI:** 10.1016/j.ibneur.2021.04.002

**Published:** 2021-05-13

**Authors:** Luis Solabre Valois, Vanilla (Hua) Shi, Paul Bishop, Bangfu Zhu, Yasuko Nakamura, Kevin A. Wilkinson, Jeremy M. Henley

**Affiliations:** School of Biochemistry, Centre for Synaptic Plasticity, Biomedical Sciences Building, University of Bristol, Bristol BS8 1TD, UK

**Keywords:** Botulinum neurotoxin, Neurotrophy, Rac1, ERK, Hippocampal neuron

## Abstract

Botulinum neurotoxins (BoNTs) are extremely potent naturally occurring poisons that act by silencing neurotransmission. Intriguingly, in addition to preventing presynaptic vesicle fusion, BoNT serotype A (BoNT/A) can also promote axonal regeneration in preclinical models. Here we report that the non-toxic C-terminal region of the receptor-binding domain of heavy chain BoNT/A (HC_C_/A) activates the small GTPase Rac1 and ERK pathway to potentiate axonal outgrowth, dendritic protrusion formation and synaptic vesicle release in hippocampal neurons. These data are consistent with HC_C_/A exerting neurotrophic properties, at least in part, independent of any BoNT catalytic activity or toxic effect.

## Highlights

• The non-catalytic, non-toxic C-terminal region of the heavy chain of BoNT/A (HC_C_/A) activates Rac1 and ERK.
• HC_C_/A promotes axonal outgrowth and dendritic filopodia.
• HC_C_/A enhances synaptic vesicle release.

## Introduction

Botulinum neurotoxins (BoNTs) comprise a ~50 kDa light chain (LC) and a ~100 kDa heavy chain (HC) connected by a disulphide bridge (Montecucco and Schiavo, 1994). The LC is the catalytic domain and the HC is responsible for BoNT delivery into neurons. BoNTs are best characterised for their blockade of synaptic transmission at the neuromuscular junction (NMJ) (Montecucco and Schiavo, 1994, Sobel, 2005). Different BoNT subtypes are referred as serotoxins due to their different reactivity to antisera (Montecucco and Rasotto, 2015). The BoNT/A serotype, which is the subject of this study, was the most toxic substance known to humankind until BoNT/H serotype was identified that has a lethal dose lower than 1 ng/kg (Barash and Arnon, 2014).

Intoxication by BoNT/A occurs in four stages: *i)* The C-terminal domain of the HC, HC_C_, binds to the cell membrane and is endocytosed into vesicles; *ii)* The N-terminal domain of the HC, HC_N_, assembles a channel in the vesicle membrane; *iii)* LC passes through the HC_N_ channel into the cell and cleaves from the HC, *iv)* the catalytic LC binds synaptosomal-associated protein of 25 kDa (SNAP-25), a critical protein required for formation of the Soluble N-ethylmaleimide-sensitive factor Attachment Protein Receptor (SNARE) complex (Schiavo et al., 1994) and completely prevents neurotransmitter release (Dong et al., 2018), but does not block endocytosis (Neale et al., 1999).

Interestingly, although BoNT/A predominantly enters neurons via synaptic vesicles at presynaptic terminals (Colasante et al., 2013) it can also gain access via binding to the fibroblast growth factor receptor 3 (Fgfr3) which undergoes dynamin-independent endocytosis (Jacky et al., 2013, Solabre Valois et al., 2020).

The extreme toxicity (LD_50_ in humans 0.1–1 ng/kg (Schiavo et al., 1994)), neuronal specificity and long persistence of BoNT/A provide opportunities for therapeutic application. Examples include treatment of muscle spasm and hypertrophy, dysphagia (swallowing difficulties), hypersialorrhea (excessive salivation), hyperhidrosis (excessive sweating), puberophonia (high-pitched speech), vocal cord paralysis, tics, motor symptoms of cerebral palsy, cancer, neuropathic pain, migraine and major depression (for reviews see (Dutta et al., 2016, Pickett and Perrow, 2011).

Because BoNTs are among the deadliest substances known, working with the full-length toxin is challenging and heavily restricted. Therefore, to eliminate toxicity while retaining the ability to investigate other BoNT actions two main options are available. One is to study the HC in isolation by removing the catalytic LC. The HC is responsible for binding to surface proteins on neurons and forming the channel that allows the LC to enter the cytosol (Koriazova and Montal, 2003). The C-terminal domain of the HC (HC_C_) has been most closely implicated in binding and internalisation (Montecucco and Schiavo, 1994) although the N-terminal domain, HC_N_, has also been reported to play a role (Ayyar et al., 2015, Montecucco, 1998).

Another option is to make the LC catalytically inactive and unable to cleave SNAP-25. The LC of BoNT/A is a zinc-dependent protease (Schiavo et al., 1992) with the Zn^2+^ ion fitting into a pocket formed by residues His223, Glu224, His227 and Glu262 (Lacy et al., 1998) with Glu224 indirectly binding zinc through a water molecule (for entire sequence see (Solabre Valois et al., 2020)). Mutation of Glu224 to a structurally similar but oppositely charged residue (E224Q), or disrupting the zinc-binding pocket with a H227Y mutation completely remove LC catalytic activity (Kukreja et al., 2007, Zhou et al., 1995). Importantly, the inactive mutants enter neurons similar to catalytically active toxins (Baskaran et al., 2013).

In addition to their remarkable toxicity BoNTs can also have positive effects on neuronal outgrowth. Motor neurons treated with BoNT/A sprout new axonal branches, an adaptive effect initially thought to be caused by the blockade of synaptic transmission (Brown et al., 1977, Holland and Brown, 1980). These new axonal branches innervate the same muscle fibres as the BoNT-intoxicated terminals, and have the same properties of calcium influx, impulse propagation and clustering of receptors at NMJ terminals as non-intoxicated control neurons (Angaut-Petit et al., 1990). BoNT/A also induces neurite sprouting in in vitro co-cultures of chick neuronal and muscle cells (Bonner et al., 1994). Moreover, BoNT/A preconditioning enhances motor axon regeneration after mechanical injury in mice and in a human stem cell model (Franz et al., 2018).

BoNT/A-induced neurite outgrowth has been proposed to be mediated by cleavage of the SNARE protein SNAP-25 (de Paiva et al., 1999). However, a subsequent study using mouse embryonic spinal cord cultures suggested that the C-terminal receptor binding domain of the heavy chain (HC_C_/A) of BoNT/A alone, which lacks any catalytic activity on SNARE proteins, is responsible for neurite outgrowth (Coffield and Yan, 2009). How these HC_C_/A-mediated neurogenic effects occur remains unclear, but an attractive hypothesis is that it involves GTPases, which are a major target for pathogens (reviewed in (Spano and Galan, 2018; Stein et al., 2012)) and are strongly implicated in neurite outgrowth (Luo et al. 1996). In particular, the small GTPase Rac1 is a substrate for ADP-ribosylation by the C3 component of botulinum toxin (Didsbury et al., 1989), and BoNT/A has been reported to upregulate expression of Rac1 and other small GTPases (Park et al., 2016).

Rac1 acts as a ‘switch’ that binds GDP or GTP depending on its activation state (Um et al., 2014). GTP-bound Rac1 interacts with effectors to modulate actin dynamics and membrane remodelling (Mackay et al., 1995, Nobes and Hall, 1995, Ridley et al., 1992). Furthermore, Rac1 plays a central role in the regeneration of injured axons. More specifically, Rac1 inhibition with the specific inhibitor NSC23766 (Gao et al., 2004) reduces axonal density and impairs functional recovery in animal models of stroke, whereas Rac1 overexpression promotes axonal regeneration and functional recovery (Liu et al., 2018).

Here we investigated the effectiveness of the catalytically inactive full-length mutant of BoNT/A, BoNT/A(0), which contains two point mutations, E224Q/H227Y (Solabre Valois et al., 2020) and HC_C_/A on neurite outgrowth. We performed experiments in neuronal stem cells and cultured rat hippocampal neurons in the presence or absence of the specific Rac1 inhibitor NSC23766 (Gao et al., 2004) to investigate the effects and mechanisms of BoNT/A actions. Our results indicate that HC_C_/A-mediated activation of Rac1 can contribute to neuronal differentiation, filopodia formation/stabilisation, axonal outgrowth and potentiate synaptic vesicle release.

## Materials and methods

### Safety

In the course of this project, no endo-positive (i.e. protease active therefore biologically hazardous) full-length botulinum neurotoxin was produced. It is important to note that in the context of BoNT production, protein activation specifically refers to hydrolysis of the peptide bond connecting LC and the HC and not to reconstitution of peptidase activity. Toxins labelled as (0) denote toxins in which protease activity has been removed by prior site-directed mutagenesis on their respective genes. This is only applicable to LC and BoNT, as HC does not present any protease action. All procedures were approved by the University of Bristol and Ipsen.

### Preparation of BoNT/A(0) and HC_C_/A

The purified BoNT proteins for use in this study were prepared and provided by Ipsen (Oxford, UK) using proprietary protocols.

### Primary neuronal cultures

All animal work was performed at University of Bristol and rats were sacrificed by schedule 1 lethal anaesthesia, following procedures in full compliance with ARRIVE guidelines and the U.K. Animals Scientific Procedures Act, 1986. Hippocampal neurons were prepared from E18 Wistar rat embryos as previously described (Gurung et al., 2018). Briefly, cells were plated on poly-L-lysine-coated culture dishes at a density of 600,000 per well (6-well plates), or on to coverslips (200,000 per coverslip) in Neurobasal medium (Gibco) containing 10% horse serum, 2% B27 (Gibco), and 2 mM glutamine, and incubated at 37 °C in humidified air supplemented with 5% CO_2_. Twenty-four hours later, the media was replaced by Neurobasal medium containing 2% B27 and 0.8 mM glutamine, and incubated at 37 °C in humidified air supplemented with 5% CO_2_ until use.

### IncuCyte imaging

To measure neurite outgrowth, we used a fully automated IncuCyte system for neurite length detection and calculating total neurite (i.e. axon and dendrite) outgrowth. Relative total neurite outgrowth values were calculated by referring each time point value of total neurite length to the total neurite length of the first image taken after cells were plated on DIV0 (i.e. the initial value is 1 for all the conditions and the value when a neurite doubles in length is 2).

### Neural stem cell cultures

NSCs were obtained from E14 Wistar rat embryos and maintained undifferentiated in NSC growth medium as described previously (Zhu et al., 2018). Briefly, the forebrains were dissected out in a petri plate containing calcium and magnesium-free Hank’s balanced salt solution (HBSS; Gibco) with 2 mM Hepes. Samples were centrifuged at 200 g for 1 min at room temperature (20–25 °C) and the supernatant was removed. 1 ml of pre-warmed StemPro Accutase (Gibco) was added to the tissue pellet and incubated at 37 °C for 10 min, and then dissociated by triturating 10 times in 10 ml of DMEM/F12 using a fire-polished pipette. The resultant cell suspension was filtered through a 70 µm cell strainer (BD Biosciences). The cells were then centrifuged at 200g for 5 min and the pellet was resuspended in 2 ml of stem cell proliferation medium comprising DMEM/F12 media supplemented with B27 supplement (Gibco), 1 × Insulin-Transferrin-Sodium Selenite Supplement (Gibco), 1 × Non-Essential Amino Acids Solution (Gibco), 20 ng/ml of both EGF and bFGF (Preprotech), and 1 × Pen-Strep (Sigma Aldrich). Cells were counted and seeded in 6-well plates or T-25 culture flasks at a density of 1 × 10^5^ ml^−1^. The medium was changed every 48 h. Neurospheres were passaged every 3–4 days when they were ~100 µm in diameter.

For experiments involving neuronal differentiation, neurospheres were dissociated into single cells with Accutase and then plated at a density of 50,000 cells per coverslip in 24-well plates coated with poly-D-lysine (Sigma-Aldrich). NSCs were incubated in neurogenic differentiation medium (the proliferation medium without EGF and bFGF but supplemented with 200 μM Ascorbic acid (Sigma Aldrich). The medium was changed every 2–3 days and the NSCs can be differentiated for up to 14 days. All neural cell transfections were performed using Lipofectamine 2000 (Invitrogen) according to the manufacturer’s instructions. Only NSCs grow/proliferate as neurospheres in suspension and 80–90% purity was confirmed with the neural stem cell marker Nestin (Zhu et al., 2018).

### Rac1 activity assay

Rac1 activation was assessed using Active Rac1 Detection Kit by Cell Signaling Technologies (CST; Cat. No. 8815), according to the manufacturer’s instructions. An aliquot of total lysate was kept as a total Rac1 control and the rest of the sample was applied to the column. Rac1 binds to an interacting protein retained on the column whereas inactive Rac1 and other proteins flow through. The total Rac1 was eluted and assessed by quantitative Western blotting together with the total Rac1 control.

### SDS-PAGE and western blotting

Samples for Western blotting were resolved by SDS-PAGE. Separated proteins were transferred to Immobilon-PVDF membrane (Merck Millipore). After protein transfer, the membrane was briefly washed with TBS-T (1x Tris Buffered Saline, 0.1% Tween), blocked in 5% non-fat milk powder for 1 h and incubated with primary antibody diluted in 5% non-fat milk powder for 1 h at RT. Following incubation, the membrane was briefly washed three times with TBS-T and incubated with HRP-conjugated secondary antibody (Sigma-Aldrich, 1:10,000) diluted in 3% non-fat milk powder for 45 min at RT. Membranes were washed three times for 5 min in TBS-T and incubated for 1 min at RT with Enhanced Chemiluminescence (ECL) substrate. Antibodies were diluted 1:1000 (Rac1 from CST, Cat. No. 8815 and phospho-ERK1/2 from Sigma, Cat. No. M7802) or 1:300 (ERK1/2 from Sigma, Cat. No. M3807). ECL substrates were from Thermo Fisher Scientific and membranes were developed using X-ray films.

### Immunocytochemistry

Cells were fixed with pre-warmed 4% PFA for 10 min at room temperature. Cells were then blocked and permeabilised with PBS containing 3% (w/v) BSA and 0.1% Triton X-100 for 20 min at room temperature. Antibodies were mixed with PBS containing 3% (w/v) BSA to their appropriate working concentrations (Tuj1 antibody (Sigma-Aldrich, Cat No. T2200) at 1:200, and AnkG antibody (Neuromab, Cat No. N106136) at 1:500. These were incubated at 4ºC overnight. Then, coverslips were washed and incubated with fluorescently labelled secondary antibodies (from Jackson ImmunoResearch) in PBS containing 3% (w/v) BSA and incubated for 1 h at room temperature. Coverslips were washed and mounted onto microscope slides and filopodia were counted as neurite protrusions with three requisites: 1) without an end wider than the body of the protrusion, 2) with a length at least twice as long as the width, 3) with a length shorter than 10 µm.

### Vesicle release

Neurons were transfected with pcDNA3-SypHy and pmCherry-C3, as a morphological marker, using Lipofectamine 2000. BoNT/A(0) and HC_C_/A were applied to the culture after transfection. Transfections and release experiments were performed as described previously (Craig et al., 2015, Girach et al., 2013, Shi et al., 2020, Tang et al., 2015). Neurons were stimulated with a MASTER 8 pulse stimulator connected to a field stimulation imaging chamber with a slotted bath. Coverslips were mounted on the chamber and sealed with grease. The chamber was fitted into a compatible holder, connected to a gravity flow perfusion system and placed on the microscope stage. Buffers were pre-warmed to 37 °C before the experiment. Stimulation protocols were as described in the literature (Burrone et al., 2006, Craig et al., 2015). Baseline recordings were measured by stimulating the chamber with 2 Hz for 30 s. Then a stimulation of 66 APs at 33 Hz was applied to release the readily-releasable pool (RRP). 10 s after this, a stimulation of 900 APs at 20 Hz was applied to release the reserve pool (RP). 120 s after the start of the experiment, ammonium chloride solution was perfused onto neurons to reveal total levels of SypHy expression in neurons. Recording was stopped 60 s after NH_4_Cl addition. 25 µM CNQX and 50 µM D-AP5 were perfused throughout the experiment. BoNT/A(0) and HC_C_/A were not present in the solutions perfused. For each cell, 15 ROIs were analysed with non-responsive ROIs being discounted. Data were first normalised to background levels (ΔF/F0) and then expressed as a percentage of fluorescence obtained after NH_4_Cl perfusion (F_max_) (Girach et al., 2013, Tang et al., 2015).

### Statistical analysis

Statistical analysis was done using MatLab and plots and graphs created using Microsoft Office Excel, as well as data handling and calculations. The following tests were carried out: Student’s *t*-test (ttest), one-way Analysis of Variance (ANOVA) (anova1) followed by multiple comparison tests (multcompare) with Tukey’s correction (Tukey-Krame). Data are presented as mean ± Standard Error of the Mean (SEM) unless indicated otherwise. In all cases, the N value refers to the number of independent experiments performed.

## Results

### HC_C_/A activates Rac1 and ERK1/2 in neurons

**Fig. 1:**
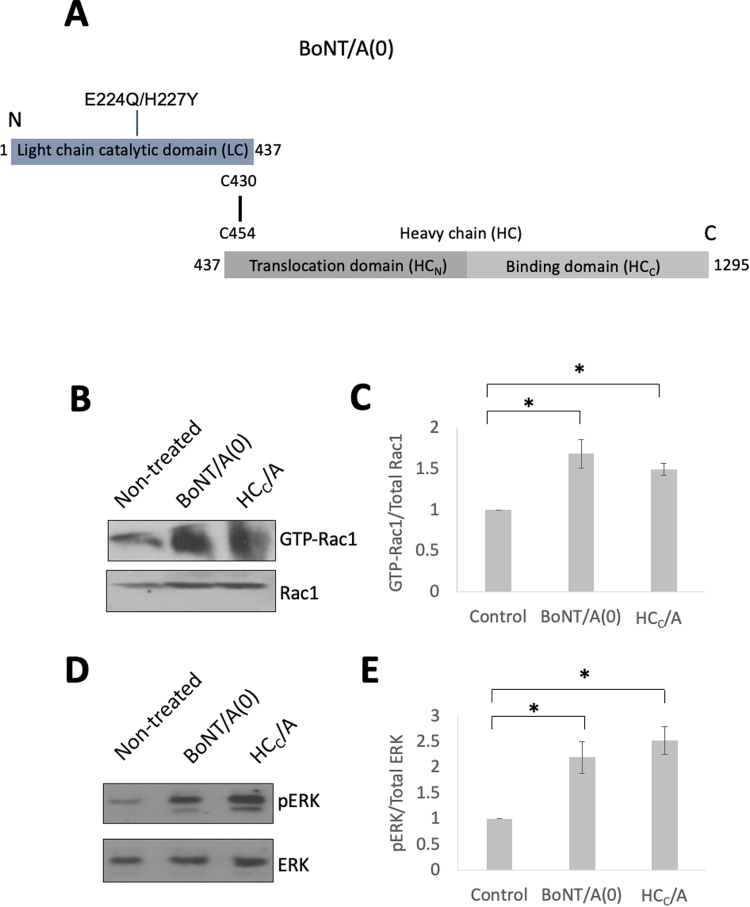
BoNT/A(0) and HC_C_/A activate Rac1 and ERK1–2**.** A) Schematic representing the domains of BONT(0), including the HC_C_/A used here. B) Representative Western blot of samples from DIV14–17 cortical neurons treated with vehicle, 25 nM BoNT/A(0) or 25 nM HC_C_/A for 30 min. Activated Rac1 was isolated using Active Rac1 Detection Kit (Cell Signalling Technologies). Isolated active Rac1 and cell lysate (for total Rac1) were then subjected to Western blotting and probed for Rac1. C) Quantification of the results in B, represented by mean values ± SEM. ANOVA followed by Tukey post hoc test. *p < 0.05. N = 3. D) Representative Western blot from DIV14–17 cortical neurons treated with vehicle, 25 nM BoNT/A(0) or 25 nM HC_C_/A for 30 min. Membranes were probed with anti-pERK and anti-ERK antibodies. E) Quantification of the results in D, represented by mean values ± SEM. ANOVA followed by Tukey post hoc test. *p < 0.05, * *p < 0.01. N = 4.

It has recently been reported that Rac1 promotes axonal regeneration by enhancing intrinsic growth (Liu et al., 2018). We therefore tested whether full-length catalytically inactive BoNT/A(0) or the HC_C_/A domain alone (schematic shown in Fig. 1A) are capable of activating Rac1. Although the IC_50_ for BoNT/A cleavage of SNAP-25 is in the pM range, BoNT/A(0) reaches a binding/internalisation plateau at ~25 nM (Vazquez-Cintron et al., 2014), a concentration widely used in the field (Harper et al., 2011, Harper et al., 2016, Restani et al., 2012, Wang et al., 2015). For consistency, in this study we used both BoNT/A(0) and HC_C_/A at the same 25 nM concentration.

Primary neurons were treated for 30 min with 25 nM BoNT/A(0) or HC_C_/A and GTP-bound (active) Rac1 was isolated from the lysate and quantified to determine the proportion of active Rac1 (Fig. 1B, C). The ratio of Rac1 bound to GTP was significantly higher in both BoNT/A(0) and HC_C_/A treated cells compared to control untreated cells (Control = 1; BoNT/A(0) = 1.69 ± 0.18; HC_C_/A = 1.49 ± 0.07; *p < 0.05 in both cases).

The extracellular signal-regulated kinase (ERK) pathway is one of the main signalling nodes for neurite outgrowth (Perron and Bixby, 1999, Tsuda et al., 2011) and neuronal regeneration (Agthong et al., 2006, Agthong et al., 2009). Rac1 activation initiates downstream ERK signalling so we next investigated the effects of BoNT/A(0) and HC_C_/A on ERK activation. Neurons were treated for 30 min with 25 nM BoNT/A(0) or HC_C_/A, lysed and active phosphorylated ERK (pERK) assessed by Western blotting. Membranes were then stripped and re-probed for total ERK (Fig. 1D, E). Consistent with the Rac1 data, the proportion of phosphorylated ERK was significantly increased in both BoNT/A(0) and HC_C_/A treated cells compared to control untreated cells. The values were Control = 1; BoNT/A(0) = 2.20 ± 0.31 (*p < 0.05); HC_C_/A = 2.52 ± 0.27 (**p < 0.01). Together, these data demonstrate that both full-length BoNT/A(0) and just the HC_C_/A binding domain effectively activate Rac1 and ERK via a pathway that does not require BoNT/A-mediated catalysis.

### HCC/A is sufficient to selectively promote Rac1-mediated axonal outgrowth

We next sought to determine the effect of HC_C_/A on neurite (nascent axon and dendrite) outgrowth, and the possible dependence of this on Rac1. To do this, we used the Rac1 specific inhibitor NSC23766, which prevents activation by GEFs (Gao et al., 2004).

**Fig. 2:**
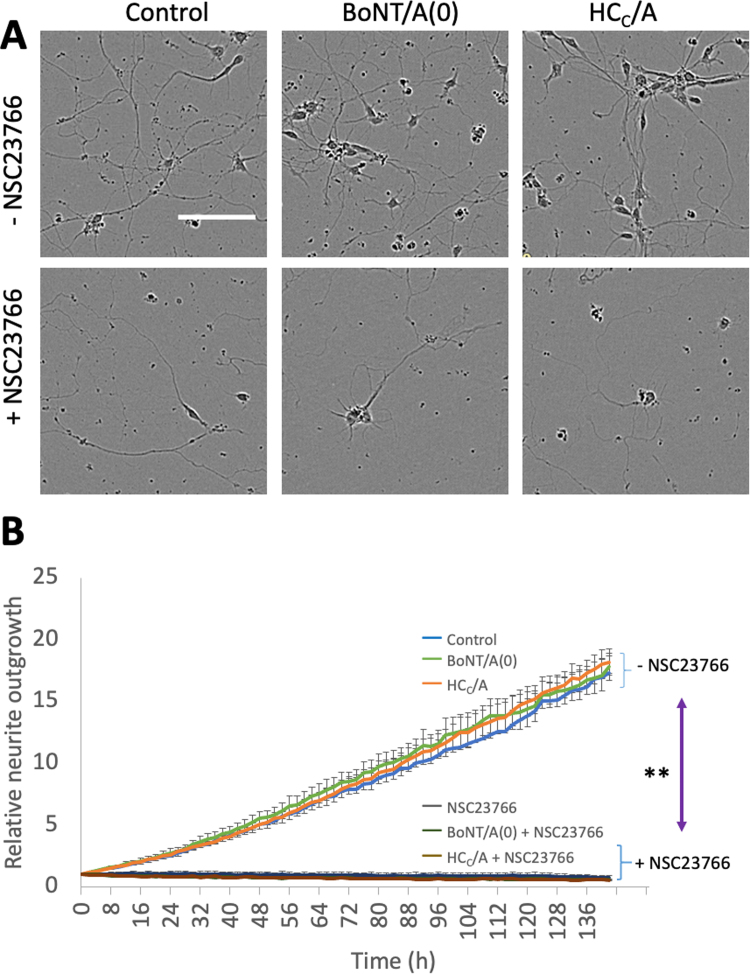
BoNT/A(0) and HC_C_/A do not globally enhance neurite outgrowth. A) Representative images of DIV7 hippocampal neurons grown in the absence or presence of 25 nM BoNT/A(0) or HC_C_/A with our without the Rac1 inhibitor NSC23766 (scale bar = 100 µM). **B)** Quantification of relative neurite outgrowth over time, represented as mean ± SEM. ANOVA followed by Tukey post hoc test, * *p < 0.01 at the end of the experiment. N = 4.

DIV0 primary neurons were plated and treated with 25 nM BoNT/A(0) or HC_C_/A in the absence or presence of 100 µM NSC23766 (Hou et al., 2014). Total neurite outgrowth was measured for 140 h using an IncuCyte live cell imaging system (Fig. 2). Neurite detection and length measurement were performed by the system and fully automated (see Fig. S1 for example validation of neurite detection). Surprisingly, in the absence of NSC23766, no significant differences were detected in neurite outgrowth between control, BoNT/A(0) and HC_C_/A groups. As expected, however, blockade of Rac1 by NSC23766 significantly reduced neurite outgrowth in all conditions (**p < 0.01; Fig. 2).

At first sight our data are in apparent contrast to a previous report that both full-length active BoNT/A holotoxin and HC_C_/A alone increase neurite outgrowth in cultured mouse embryonic spinal cord neurons (Coffield and Yan, 2009). The maximum HC_C_/A concentration used by Coffield and Yan was 10 nM, while our working concentration was 25 nM. More importantly, however, they used DRG sensory neurons rather than the hippocampal pyramidal neurons investigated here. This is significant because DRG neurons have a pseudo-unipolar morphology, containing only a single bifurcating axonal process (Nascimento et al., 2018). Thus, the fact that neither BoNT/A(0) nor HC_C_/A induced an increase in total neurite outgrowth in our experiments could be attributable to the differences in morphology of the neuronal types investigated. Indeed, BoNT/A is known to induce axonal sprouting at motor nerve terminals (Pamphlett, 1989), and Coffield and Yan’s images (Coffield and Yan, 2009) show most outgrowth corresponded to thin neurites with a terminal conical structure, suggestive of axons.

**Fig. 3:**
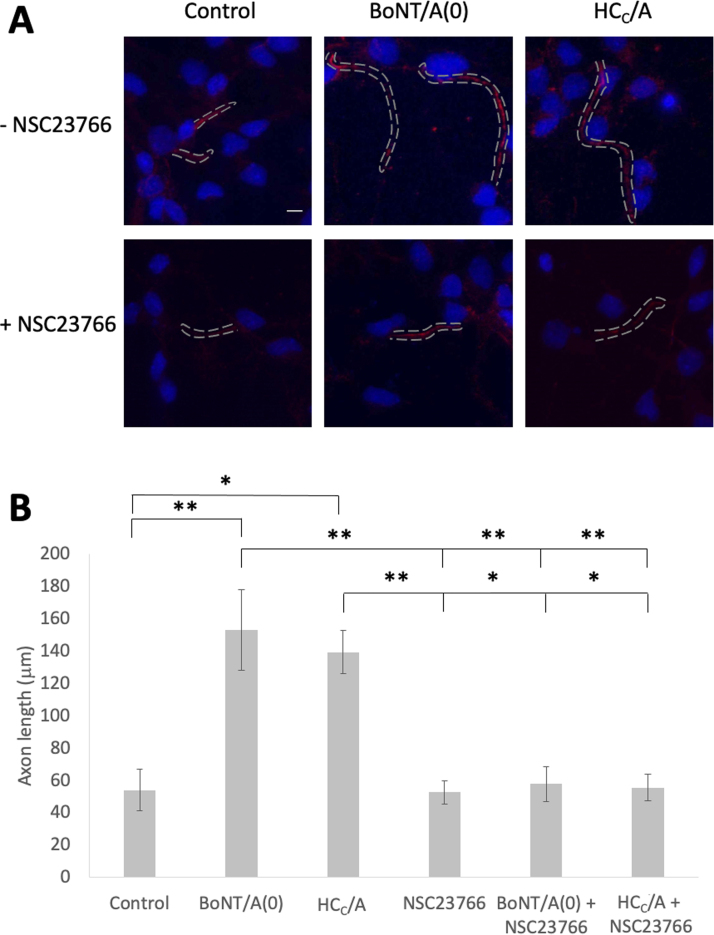
BoNT/A(0) and HC_C_/A induce axon outgrowth in a manner that requires Rac1 activity. A) Representative images of DIV2 hippocampal neurons grown with 25 nM BoNT/A(0) or HC_C_/A in the absence or presence of 100 µM NSC2736. Axons with positive AnkG staining are shown as red and outlined by dotted lines. Blue signal corresponds to nuclei (DAPI). Scale bar = 10 µm. B) Quantification of axonal outgrowth, represented as mean ± SEM. ANOVA followed by Tukey post hoc test, *p < 0.05, * *p < 0.01. Analysis of at least 3 cells per coverslip using coverslips from 3 independent cultures per condition. N = 3.

Therefore, because most of the neurites in hippocampal neurons develop into dendrites we wondered whether BoNT/A might have a selective effect on axonal outgrowth, which was obscured in our IncuCyte imaging. To examine this directly, we selectively monitored axonal outgrowth in our very young hippocampal cultures. DIV1 hippocampal neurons were plated and treated with 25 nM BoNT/A(0) or HC_C_/A in the absence or presence of 100 µM NSC23766. At DIV2, cells were fixed and stained with the axonal initial segment marker Ankyrin-G (AnkG) to identify axons (Fig. 3). In developing neurons, AnkG can be stained as early as DIV1 and stains all of the nascent axon (Fréal et al., 2016, Kuijpers et al., 2016).

BoNT/A and HC_C_/A treated neurons exhibited significantly increased axonal length compared to untreated controls. The axonal length values were: control = 53.9 ± 12.8 µm; BoNT/A(0) = 153.3 ± 25.0 µm (**p < 0.01 compared to control) and HC_C_/A = 139.3 ± 13.3 µm (*p < 0.05 compared to control). However, in the presence of NSC23766, these values were reduced to control levels: 52.6 ± 7.4 µm; BoNT/A(0) = 57.7 ± 11.0 µm; HC_C_/A = 55.6 ± 8.34 µm. These data, together with those of Coffield and Yan’s images (Coffield and Yan, 2009), suggest that HC_C_/A selectively promotes axonal outgrowth, and that this effect requires activation of Rac1.

### HC_C_/A promotes the formation of dendritic protrusions

**Fig. 4:**
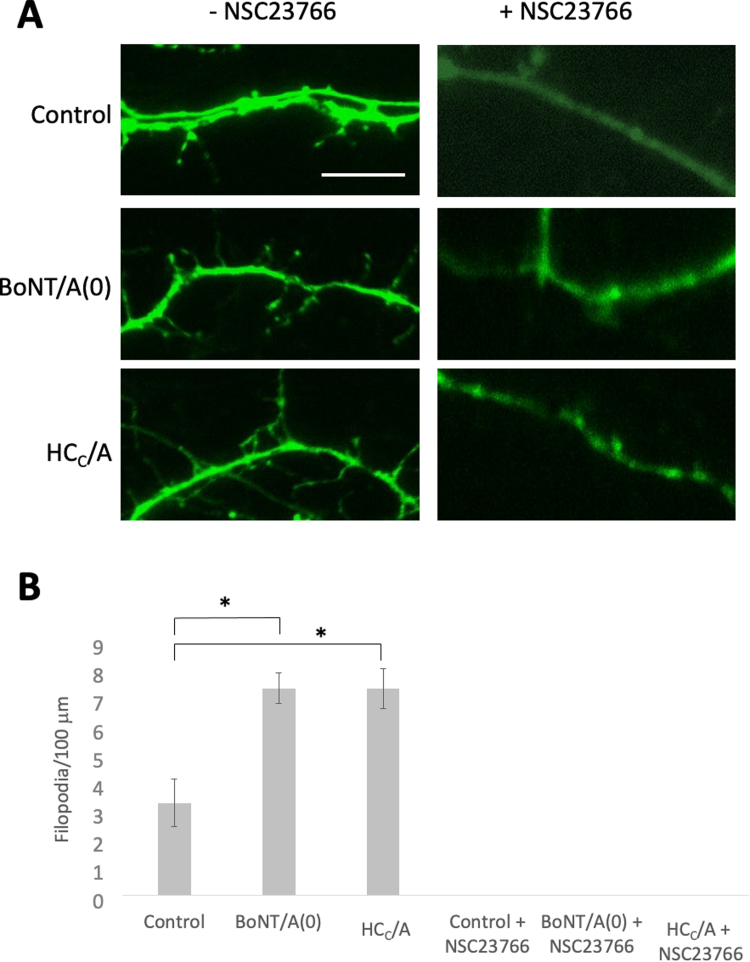
BoNT/A(0) and HC/A increase the number of dendritic protrusions. A) Representative images of DIV14–17 hippocampal neurons transfected with a plasmid encoding eGFP and treated for 3 days with 25 nM BoNT/A(0) or HC_C_/A in the absence or presence of 100 µM NSC23766. B) Quantification of the number of protrusions per unit of neurite length, represented as mean values ± SEM. ANOVA followed by Tukey post hoc test. *p < 0.05. Analysis of at least 3 cells per coverslip using coverslips from 3 independent cultures per condition. N = 3. Scale bar = 10 µm.

Although our data suggest that HC_C_/A does not significantly alter nascent dendrite outgrowth we wondered if it affects formation of dendritic protrusions, i.e. filopodia that can mature into dendritic spines (Ziv and Smith, 1996), and if this also requires Rac1 activity (Yoshihara et al., 2009). To explore this possibility, we treated DIV14–15 hippocampal neurons with 25 nM BoNT/A(0) or HC_C_/A for 3 days in the absence or presence of 100 µM NSC23766 (Fig. 4). Neurons were transfected with eGFP prior to the treatment to allow visualisation of neuronal morphology. For these experiments we counted protrusions from the main body of the dendrite, meaning that both filopodia and early dendritic spines, which begin to appear at this developmental stage in our cultures, are included (Ashby et al., 2004, Martin et al., 2007). In our control neurons there were 3.31 ± 0.86 protrusions/100 µm length of dendrite. In toxin-treated cells these values were significantly higher with 7.42 ± 0.55 protrusions/100 µm for BoNT/A(0)-treated cells and 7.40 ± 0.72 protrusions/100 µm for HC_C_/A-treated cells (*p < 0.05 compared to control in both cases). No well-defined protrusions at all were observed for NSC23766-treated cells under any condition. Thus, analogous to our results for axonal outgrowth, these data suggest that HC_C_/A promotes the formation of dendritic protrusions, and that this process too requires Rac1 activity.

### HC_C_/A potentiates release of the reserve pool of synaptic vesicles

**Fig. 5:**
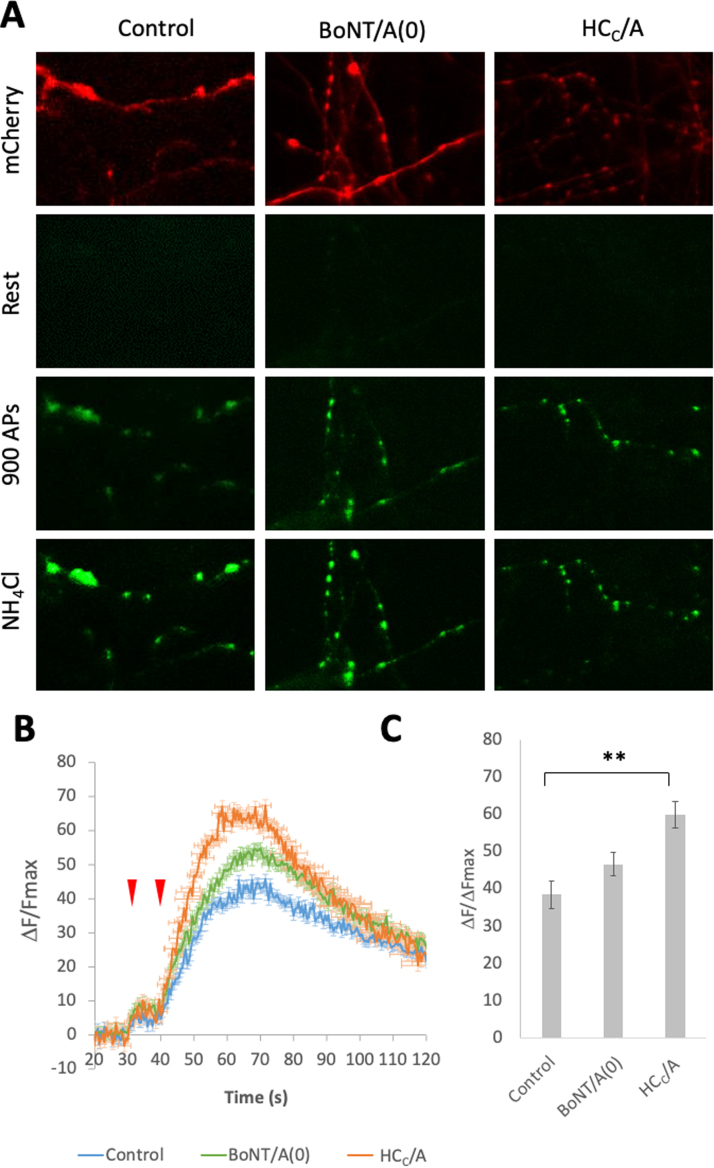
HC_C_/A promotes release of the reserve pool of synaptic vesicles, A) Representative images of from DIV14–16 hippocampal neurons transfected with SypHy and mCherry. Cells were treated for 3 days with 25 nM BoNT/A(0) or HC_C_/A and, after baseline recordings, stimulated to release the readily-releasable pool of synaptic vesicles (66 APs at 33 Hz) followed, 10 s later, by a stimulation of 900 APs at 20 Hz to release the reserve pool. The maximal fluorescence was revealed by NH_4_Cl, which temporally equilibrates all intracellular compartments to the extracellular pH 7.4. B) Quantification of the SypHy signal, represented as mean ± SEM. Red arrows indicate stimulations described in A. C) Quantification of reserve pool release showing the average ΔF/F_max_ comprising values from 10 s after the second stimulation until the peak begins to subside at 70 s. Analysis of at least 3 cells per coverslip using coverslips from 3 independent cultures per condition. * *p < 0.01. N = 3.

Given that our data indicate that BoNT/A(0) and HC_C_/A promote both axonal outgrowth and increase the number of dendritic protrusions, we next investigated other possible neurotrophic effects of BoNT, such as enhancement of presynaptic neurotransmitter release (Fig. 5).

Hippocampal neurons were treated with 25 nM BoNT/A(0) or HC_C_/A for 3 days and the presynaptic vesicle release at individual boutons induced by electrical stimulation measured using synaptophysin pHluorin (SypHy; (Craig et al., 2015, Girach et al., 2013, Shi et al., 2020, Tang et al., 2015). The SypHy assay uses a pH-sensitive fluorophore reporter tagged to the extracellular region of synaptophysin to detect synaptic neurotransmitter release. At rest, SypHy fluorescence is quenched due to the acidic conditions inside the lumen of the vesicle. However, upon stimulation, vesicular fusion to the plasma membrane exposes the reporter to the neutral extracellular solution, enhancing fluorescence (Burrone et al., 2006).

BoNT/A and HC_C_/A both increased release from the reserve pool of synaptic vesicles with peak values of: control = 38.5 ± 3.7; BoNT/A(0) = 46.7 ± 3.3; HC_C_/A = 60.0 ± 3.6 (Fig. 5). However, the increase in synaptic vesicle release was only significantly different between control and HC_C_/A groups (**p < 0.01) with no statistical difference between control and BoNT/A(0), or BoNT/A(0) and HC_C_/A. While the reason for these differences is currently unclear, our data suggest that HC_C_/A alone is sufficient to enhance presynaptic release.

### BoNT/A(0) promotes neurogenesis in a manner dependent on Rac1

**Fig. 6:**
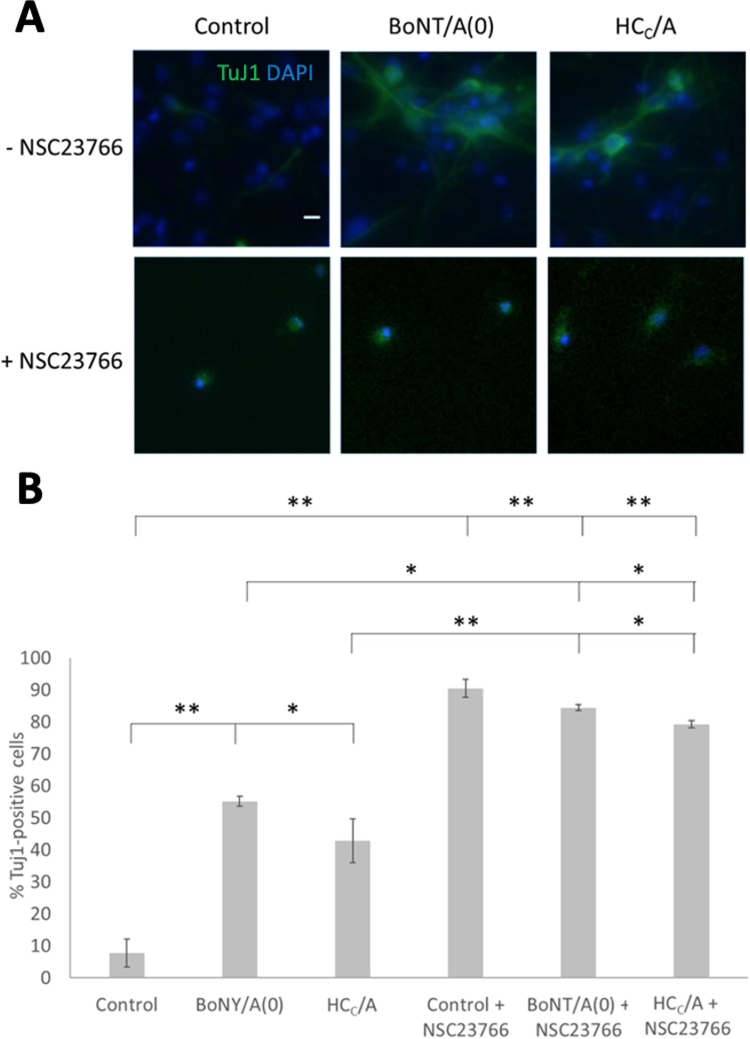
BoNT/A(0), HC_C_/A and NSC23766 increase NSC neuronal differentiation. A) Representative images of DIV10 NSCs grown in non-differentiated conditions in the absence or presence of 25 nM BoNT/A(0) or HC/A, with or without 100 µM NSC23766, and stained with anti-Tuj1 (green) as a marker for differentiation and DAPI (blue) to stain nuclei. Scale bar = 10 µM. B) Quantification of the proportion of Tuj1-positive cells represented as mean ± SEM. ANOVA followed by Tukey post hoc test. *p < 0.05, * *p < 0.01. Analysis of multiple fields of view per coverslip using coverslips from 3 independent cultures per condition. N = 3.

Our results so far demonstrate that BoNT/A possesses a number of neurotropic properties, namely the promotion of axonal growth, of dendritic filopodia and enhancement of neurotransmitter release. We therefore wondered whether BoNT/A was also capable of promoting neuronal differentiation. To investigate this, we examined the effects of HC_C_/A on directing neurogenesis of neural stem cells (NSCs) (Song et al., 2012, Yuan et al., 2011). Neuronal differentiation of NSCs was assessed by measuring expression of the neuron-specific marker Class III β-tubulin (Tuj1) (Jiang and Oblinger, 1992, Katsetos et al., 2007). Cells were plated after neurosphere dissociation and treated with 25 nM BoNT/A(0) or HC_C_/A in the absence or presence of NSC23766. On DIV10, cells were fixed and stained with an antibody targeting Tuj1 (Fig. 6A).

Data were first analysed by examining the proportion of Tuj1-positive cells in each condition. Tuj1-positive cells made up 7.70 ± 6.34% under control conditions in the absence of NSC23766, indicating minimal spontaneous differentiation. Both BoNT/A(0) and HC_C_/A dramatically promoted differentiation, with values of 55.2 ± 3.2% (**p < 0.01) and 42.7 ± 3.7% (*p < 0.05), respectively. However, for NSC23766-treated conditions, the percentage of differentiated cells went up to 90.5 ± 9.5% for the control, 87.8 ± 6.2% for BoNT/A(0)-treated cells and 86.3 ± 3.2% for HC_C_/A-treated cells (Fig. 6A, B). These values are significantly higher than the respective conditions without NSC2733 treatment. Importantly, however, the pro-differentiation effect of BoNT/A(0) and HC_C_/A were not observed in the presence of NSC23766. Nonetheless, these surprising results could be interpreted to suggest that either inhibition by NSC23766, or activation of Rac1 by BoNT/A(0) and HC_C_/A, or both, induce NSC differentiation.

We note, however, the interpretation of the effect of NSC23766 is confounded by the fact that NSC23766 exerted a strong anti-proliferative effect on NSCs (Fig. S2A)**.** The cell count per 10,000 µm^2^ (100 ×100 µm) was 11.6 ± 0.4 for the control (a total of 217 cells), 16.8 ± 4 (220 cells) for the group treated with BoNT/A(0) and 18.5 ± 3.8 (247 cells) for the group treated with HC_C_/A. In presence of NSC23766, the number of cells per field was significantly reduced to 2.77 ± 0.86 (*p < 0.05 compared to the corresponding NSC23766-lacking group, 52 cells), 1.65 ± 0.14 (**p < 0.01, 31 cells) and 1.28 ± 0.24 (**p < 0.01, 34 cells). Thus, we conclude that the NSC23766-induced reduction of the cell count was responsible for the abnormal increase in the percentage of differentiation, and, since most surviving cells were differentiated, this suggests NSC23766 specifically depletes undifferentiated NSCs.

We also analysed the number of Tuj1-positive cells per 10,000 µm^2^ unit area (Fig. S1B). Both BoNT/A(0) and HC_C_/A increased the number of Tuj1-positive cells per unit area, and this effect was lost in the presence of NSC32766. For the control, 0.85 ± 0.70 cells were Tuj1-positive. BoNT/A(0) significantly increased the number of Tuj1-positive cells per unit area, to 6.40 ± 0.24 (**p < 0.01 compared to the control). HC_C_/A caused a similar effect, with 7.63 ± 1.1 Tuj1-positive cells per field (**p < 0.01 to the control). For NSC23766-treated groups, these values were 2.34 ± 0.44, 1.39 ± 0.14 and 1.01 ± 0.19 for the control, BoNT/A(0)-treated and HC_C_/A-treated conditions, respectively.

In combination, our data suggest that Rac1 activation by BoNT/A(0) and HC_C_/A induce NSC differentiation and that, in the absence of Rac1 activity, the neurogenic effect of BoNT is lost. Furthermore, we conclude that Rac1 is fundamental for NSC growth in vitro and its inhibition results in arrest of the cell cycle, as noted for colon cancer cells (Huang et al., 2018), and/or cell death by apoptosis (Stankiewicz et al., 2015).

## Discussion

Here we show that the HC_C_/A heavy chain binding domain affects a range of neuronal properties, including axonal and filopodial outgrowth, synaptic vesicle release and NSC differentiation, with no requirement for BoNT catalytic activity. Moreover, we demonstrate that these actions require activation of Rac1.

### Axonal outgrowth

We did not detect any effect of BoNT/A or HC_C_/A on total neurite outgrowth. However, using AnkG as a marker for neurites destined to be axons (Fréal et al., 2016, Kuijpers et al., 2016), we demonstrate that HC_C_/A selectively promotes axonal outgrowth in hippocampal neurons, and that this effect is lost upon inhibition of Rac1. These data are consistent with the finding that overexpression of human Rac1 in mouse Purkinje cells induces axonal, but not dendritic, outgrowth (Luo et al., 1996). Our results also agree with a study in DRG neurons where BoNT/A concentrations in the range 0.01 – 10 nM were reported to stimulate neuritogenesis (Coffield and Yan, 2009). Moreover, those authors observed similar results with the isolated HC_C_/A, suggesting that neuritogenesis could be initiated solely by the binding actions of BoNT/A to neurons.

We also show that BoNT/A(0) and HC_C/_A activate ERK1/2. In macrophages BoNT/A stimulates ERK phosphorylation via upregulation of Toll-like receptor 2 (TLR2) (Kim et al., 2015), and TLR2 promotes phosphorylation of ERK in neurons (Paulino et al., 2011). Therefore, it remains unclear if ERK1/2 activation is directly downstream of Rac1 activation. Nonetheless, taken together, our data indicate that the axonal growth promoting effects of BoNT/A(0) or HC_C_/A preferentially act on nascent axons in a Rac1-dependent manner.

### Dendritic protrusions

BoNT(0)/A and HC_C_/A induce dendritic protrusion formation in our cultures, and this effect is abolished by the Rac1 inhibitor NSC23766. It has been reported previously that Rac1 can enhance dendritic filopodia formation (Alvarez Julia et al., 2016, Galic et al., 2014) and their maturation into functional spines (Fiuza et al., 2013, Tashiro and Yuste, 2004). It should be noted, however, that long-term treatment with NSC23766 will inhibit all Rac1 activity and impair many cellular functions. For example, NSC23766, can induce apoptosis (although demonstrated at 200 µM, rather than the 100 µM used here (Stankiewicz et al., 2015)) and dendritic protrusions are pruned through pathways shared with apoptosis (Meng et al., 2015a) so we cannot completely exclude the possibility that the inhibition of filopodia formation by NSC23766, could be due to initiation of apoptotic, or other, pathways rather than a direct Rac1-mediated effect on filopodia formation. Notwithstanding these potential caveats, our data demonstrate a positive effect of HC_C_/A on the formation of dendritic protrusions.

### Vesicle release

Surprisingly, while HC_C_/A significantly increased synaptic vesicle release from the presynaptic terminal, BoNT/A(0) did not. Importantly, unlike HC_C_/A, full-length BoNT/A(0) can deliver LC/A(0) to the presynaptic terminal (Vazquez-Cintron et al., 2014) where, despite being catalytically inactive, LC/A(0) binds to SNAP-25 (Pellett et al., 2011), possibly interfering with SNARE complex function. While this non-catalytic inhibitory interaction could prevent the enhanced synaptic vesicle release observed with HC_C_/A, the reasons for the differential effects of HC_C_/A and BoNT/A(0) on release are unclear and require further investigation. We postulate that Rac1 could be involved in the HC_C_/A-mediated potentiation of transmitter release. Consistent with this idea, loss of Rac1 impairs synaptic plasticity and spatial learning (Haditsch et al., 2013) and it also facilitates Ca^2+^-dependent exocytosis (Li et al., 2003). However, we were unable to test this hypothesis because the Rac1 inhibitor NSC23766 compromises neuronal viability and function. Indeed, inactivation of Rac1 by knock-out or NSC23766 treatment elicits apoptosis (Stankiewicz and Linseman, 2014; Hua et al., 2015).

### Neurogenesis

In addition to structural and functional changes in neurons, we addressed the question of the potential influence of BoNT on neurogenesis. Our experiments indicate that BoNT/A(0) and HC_C_/A promote NSC differentiation, and that this effect is blocked by Rac1 inhibition. These findings are consistent with the previously reported roles of Rac1 in promoting neurogenesis evoked through nerve growth factor (NGF) (Suzukawa et al., 2000) and by learning (Haditsch et al., 2013).

Conversely, inhibition of Rac1 or downstream Notch2 signalling pathways have been suggested to enhance NSC differentiation (Meng et al., 2015b). However, this is likely due to the arrest of cell proliferation rather than a direct neurogenic effect. Indeed, in our cells, Rac1 inhibition arrested proliferation. Rac1 elicits neuroprotective effects (Stankiewicz et al., 2015) and an important factor in NSC proliferation is that low density leads to spontaneous differentiation (Cha et al., 2017). We propose that the enhanced differentiation we observed upon Rac1 inhibition corresponds to an apoptotic effect followed by spontaneous differentiation caused by the low density of culture, but further work will be required to fully test this hypothesis.

## Conclusion

In conclusion, we have demonstrated that BoNT/A elicits neurotrophic effects via HC_C_/A-mediated activation of Rac1. Properly administered, BoNT/A has already been shown to be a safe drug and, indeed, it induces sprouting in nerves of patients suffering from Parkinson’s disease and amyotrophic lateral sclerosis (Kulshreshtha et al., 2020, Moller et al., 2011, Ruiz-Roca et al., 2019, Vazquez-Costa et al., 2016). Clearly, caution must be used when comparing the sprouting response at the neuromuscular junction in vivo with neurite outgrowth from cultured neurons. Motor nerve terminal sprouting is well established to result from blockade of neurotransmitter release (Comella et al., 1993, Raghunath et al., 2008, Thesleff et al., 1990) and is part of a neurodevelopmental programme that provides new active extra-junctional synapses at the NMJ that requires perisynaptic Schwann cells (Son and Thompson, 1995). Nonetheless, our data suggest that, in addition to the toxic catalytic activity of BoNT/A blocking neurotransmitter release, activation of Rac1 and ERK pathways by the HC_C_/A may have important roles in neurogenesis in cultured pyramidal neurons.

## CRediT authorship contribution statement

**Luis Solabre Valois:** Conceptualization, Data curation, Formal analysis, Investigation, Methodology, Writing - original draft, Writing - review & editing. **Kevin A. Wilkinson:** Conceptualization, Writing - review & editing, Funding acquisition, Resources, Writing - original draft, Writing - review & editing. **Jeremy M. Henley:** Conceptualization, Funding acquisition, Writing - original draft, Writing - review & editing. **Vanilla (Hua) Shi:** Data curation, Investigation, Methodology, Writing - review & editing. **Yasuko Nakamura:** Resources, Writing - review & editing. **Paul Bishop:** Investigation, Methodology, Resources, Writing - review & editing. **Bangfu Zhu:** Writing - review & editing,

## Conflicts of interest

LSV received a collaborative scholarship from Ipsen to fund his PhD. None of the workers at Ipsen participated in the design, performance or analysis of the experiments, or in writing the manuscript, but they did provide facilities and reagents necessary for completion of the work.
