# Supplementary figures and images for: Neurotrophic effects of Botulinum neurotoxin type A in hippocampal neurons involve activation of Rac1 by the non-catalytic heavy chain (HCC/A)
Source: IBRO Neurosci Rep. 2021 May 13;10:196–207. doi: 10.1016/j.ibneur.2021.04.002 (PMC8143998; doi:10.1016/j.ibneur.2021.04.002)

**Supplementary Material**

**
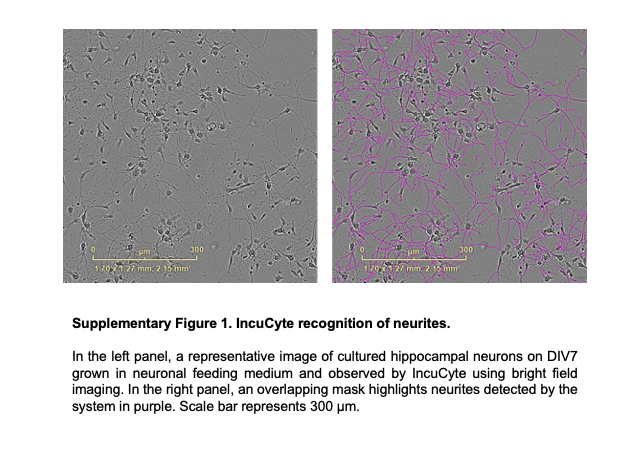
**

**
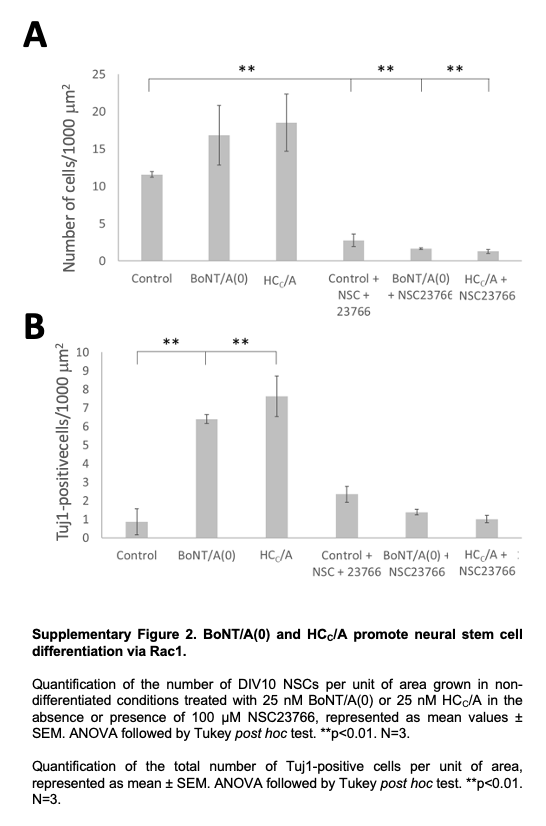
**

Supplement: Supplementary file 1 — Supplementary material [file mmc1.docx]
